# Renal Primitive Neuroectodermal Tumor: A Case Report

**DOI:** 10.1097/MD.0000000000002304

**Published:** 2015-12-11

**Authors:** Cheng Yang, Hanjiang Xu, Jun Zhou, Zongyao Hao, Jianzhong Wang, Changmin Lin, Li Zhang, Xia Zhu, Chaozhao Liang

**Affiliations:** From the Department of Urology (CY, HX, JZ, ZH, JW, LZ, CL), The First Affiliated Hospital of Anhui Medical University, Hefei; Department of Urology (CL), The Central Hospital of Maanshan, The Affiliated Hospital of Wannan Medical College, Maanshan; and Department of Pathology (XZ), The First Affiliated Hospital of Anhui Medical University, Hefei, China.

## Abstract

Primitive neuroectodermal tumor (PNET) is a malignant small round cell tumor and typically arises from bone or soft tissue in adolescents and young adults. Renal PNET is extraordinarily rare and exhibits highly aggressive biological behavior with poor prognosis.

We present here a new case of renal PNET in a 31-year-old female. The patients were referred to our hospital because of left flank pain with nausea and vomiting for 1 week. A computed tomography scan revealed a 14.7 × 12.7 cm well-defined, unevenly mass lesion with both solid and cystic components and the tumor was not enhanced uniformly.

A preoperative diagnosis of cystic renal cell carcinoma and urinary tract infection was made. The patient undergone anti-inflammatory therapy followed by a left radical nephrectomy. Taken with morphological pattern and immunohistochemical markers, a diagnosis of renal PNET was made. Two cycles of combined chemotherapy were executed. At the 14-month follow-up, no evidence of metastasis or recurrence was indicated.

This case reminds clinicians that for adolescents and young adults with a suspicious renal mass, a diagnosis of renal PNET should be always considered. An initial surgery followed by radiotherapy and chemotherapy is suggested for the therapeutic management.

## INTRODUCTION

Primitive neuroectodermal tumor (PNET) is a malignant small round cell tumor and typically arises from bone or soft tissue in the trunk or axial skeleton in adolescents and young adults. Localization of PNET in genitourinary system such as kidney, bladder, and prostate is rare.^1–4^ Renal PNET exhibits highly aggressive biological behavior with poor prognosis and till date <100 cases have been reported.^5^ The diagnosis of renal PNET is mainly based on histopathology and immunohistochemistry, supported by cytogenetic analysis, thus preoperative diagnosis is challenging. For instance, formation of the Homer-Wright rosettes and identification of CD99 and Fli-1 expression are critical evidences for diagnosis.^3,6,7^ In addition, cytogenetic detection of EWL/FLI-1 fusion protein is strongly supportive for final diagnosis.^8,9^ Treatment strategies of renal PNET is multimodal, including surgery, adjuvant chemotherapy, and radiotherapy. Owing to the high aggressiveness and strong tendency of local recurrence and remote metastases, the overall prognosis of renal PNET is poor. The patients reveal a 5-year disease-free survival of 45% to 55%.^10^ We present here the clinical, histopathological, immunohistochemical, cytogenetic findings, and treatment outcomes in a new case of renal PNET in a 31-year-old female.

## CONSENT

Informed consent was signed by the patient for the publication of this report and related images.

## CASE PRESENTATION

A 31-year-old female patient was referred to our hospital because of left flank pain with nausea and vomiting for 1 week. History of hematuria was absent. Apart from appendectomy that was performed 5 years before, no accompanying disease was present. A physical examination revealed pain on percussion on left lumbar region.

**FIGURE 1:**
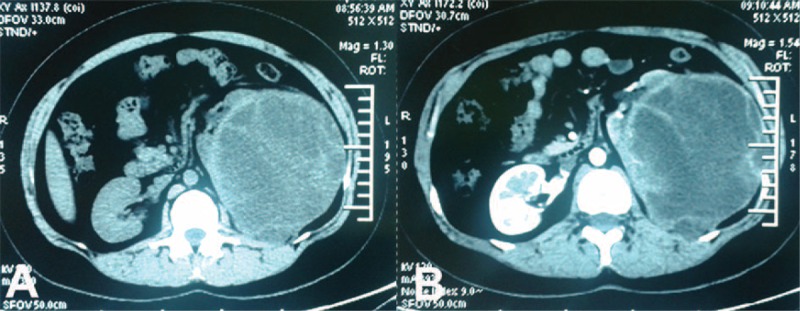
(A) Computer tomography (CT) scan revealed a 14.7 × 12.7 cm well-defined, unevenly mass lesion with both solid and cystic components. (B) The tumor was not enhanced uniformly.

All of her laboratory data were within normal limits except for a dramatically increased number of leukocytes in urine (2724/μL). On ultrasonographic examination a large heterogeneous echo pattern mass without blood flow signal in left kidney area was noticed. A computed tomography (CT) scan revealed a 14.7 × 12.7 cm well-defined, unevenly mass lesion with both solid and cystic components and the tumor was not enhanced uniformly (Fig. 1). Renogram indicated severely impaired glomerular filtration rate (14.1 mL/min) of the left kidney, and the glomerular filtration rate of right kidney was within normal range. No positive findings were revealed from chest x-ray.

Taken together with the result of physical examination, imaging, and laboratory tests, a preoperative diagnosis of cystic renal cell carcinoma and urinary tract infection was made. Anti-inflammatory therapy followed by a left radical nephrectomy was then carried out.

**FIGURE 2:**
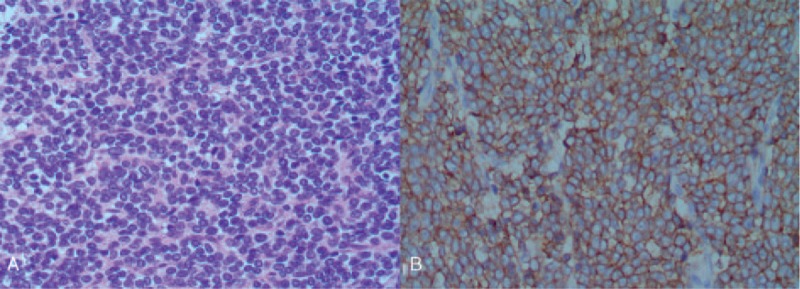
(A) Hematoxylin-eosin (H&E) staining showed small uniform round cells with scanty cytoplasm and hyperchromatic nuclei (×200). (B) Immunohistochemical staining showed CD99 was ubiquitously expressed (×200).

Macroscopically, a tumor measuring 13 × 8 × 6 cm in maximum diameter was noticed in the mid pole of left kidney. The cut section of the mass was grayish white with confined areas of solid and cystic components. The renal vein, urethra, lymph nodes, upper, and lower poles of the kidney were negative for malignancy. Histologically, small round undifferentiated tumor cells with scant cytoplasm and hyperchromatic nuclei were observed infiltrating into renal parenchyma (Fig. 2A). Formation of Homer-Wright rosettes was identified in several areas. Immunohistochemically, the tumor cells revealed strong expression of CD99 and Fli-1^3,6^ (Fig. 2B). A minor proportion of the cells was positive for NSE, Ki-67, and CD117. The tumor was negative for CK, CD56, Syn, CgA, LCA, CK7, RCC, CD10, S-100, Des, EMA, and SMA. Taken with morphological pattern and immunohistochemical markers, a diagnosis of PNET was made. In addition, the presence of EWS/FLI-1 fusion products was detected by fluorescence in situ hybridization, confirming the diagnosis.^8,9^

The patient undergone 2 cycles of chemotherapy with VAC-IE protocol using vincristine, adriamycin, cyclophosphamide, alternating with ifosfamide and etoposide within 2 weeks of interval. The patient rejected receiving chemotherapy because of the severe side effect and remained free of recurrence or metastasis for 14 months.

## DISCUSSION

PNET was initially identified as member of “small round tumors” by Arthur Purdy Stout in 1918.^8^ It typically occurs in bone or soft tissue and rarely reported a renal localization. The first case of renal PNET was reported by Mor et al in 1994.^11^ Renal PNET manifests in adolescents and young adults, with an average age of 27 and a slight male predominance.^12,13^ The presenting symptoms of renal PNET are nonspecific, including flank pain, hematuria symptoms related to genitourinary infections.^14–16^ Laboratory parameters are mostly with in normal range except altered level of lactate dehydrogenase and neuron specific enolase in several reports.^13,16^ CT images of renal PNET are also without characteristic signs, commonly revealing solitary, large, ill-defined, irregular, or lobulated heterogeneous mass.^17,18^ Therefore, the diagnosis of renal PNET is mainly based on the combination of histopathology and immunohistochemistry, supported by cytogenetic study. Macroscopically, the cutting surface of the tumor was often grayish white, with various regions of necrosis and hemorrhage.^19^ Microscopically, renal PNET is characterized by monotonous proliferation of immature and small round cells. Formation of the Homer-Wright rosettes is typical feature of renal PNET.^12^ Immunohistochemically, renal PNET is frequently positive for CD99, a vital clue for the diagnosis.^3,6,20^ In addition, almost two-thirds reveal Fli-1 expression.^7^ Other markers such as vimentin, cytokeratin, NSE, and S-100 have also been detected.^14,16^ Cytogenetic study of renal PNET is a supportive method to make the final diagnosis. Chromosomal translocation t(11;22)(q24;q12) usually can be identified in renal PNET, resulting in the formation of the EWL/FLI-1 fusion protein.^8,9^ Furthermore, neural neurosecretory granules in renal PNET have also been detected by electron microscopy.^21,22^

Treatment strategies of renal PNET are multimodal, including surgery, adjuvant chemotherapy, and radiotherapy. Patients usually present a large mass at diagnosis and the diameter of tumor is frequently >10 cm.^4,16^ In addition, owing to the difficulty in preoperative diagnosis, a neo-adjuvant chemotherapy before surgery is not applicable.^4^ Therefore, majority of the patients undergone radical nephrectomy as the initial treatment, which indicated survival advantage.^23^ Nephron-sparing surgery performed in early renal PNET has also been reported and indicated a favorable outcome.^24^ The current adjuvant chemotherapy regimens of renal PNET include vincristine, doxorubicin, and cyclophosphamide substituted by ifosfamide or etoposide.^25^ Importantly, the high rate of noncompliance with chemotherapy has been shown in previous report as well as in the current case, which should be considered while applying the therapy.^4^ In case of the local invasion of perinephric tissue or positive surgical margins, postoperative radiotherapy is also recommended.^13^ Owing to the high aggressiveness and strong tendency of local recurrence and remote metastases to organs such as lungs, liver, and bone, the overall prognosis of renal PNET is poor. The patients reveal a 5-year disease-free survival of 45% to 55%, whereas patients with metastasis indicated median relapse-free survival of only 2 years.^10^

In general, renal PNET is an extraordinarily rare disease and with highly aggressiveness and poor prognosis. In adolescents and young adults with a suspicious renal mass, a diagnosis of renal PNET should be always considered. The golden standard to diagnosis renal PNET is based on histologic and immunohistochemical features, supported by cytogenetic analysis. Multimodality treatment consisting of surgery, chemotherapy and radiotherapy is recommended to manage the disease.
